# Red Blood Cell Transfusion in the Emergency Department: An Observational Cross-Sectional Multicenter Study

**DOI:** 10.3390/jcm10112475

**Published:** 2021-06-02

**Authors:** Olivier Peyrony, Danaé Gamelon, Romain Brune, Anthony Chauvin, Daniel Aiham Ghazali, Youri Yordanov, Aude Arsicaud, Pauline Gilleron, Sonja Curac, Marie-Caroline Richard, Anne-Laure Feral-Pierssens, Barbara Villoing, Sébastien Beaune, Hélène Goulet, Jean-Paul Fontaine, Anne François, France Pirenne

**Affiliations:** 1 Emergency Department, Hôpital Saint-Louis, Assistance Publique-Hôpitaux de Paris, 1 Avenue Claude Vellefaux, 75010 Paris, France; danaegamelon@gmail.com (D.G.); romain.brune@gmail.com (R.B.); jean-paul.fontaine@aphp.fr (J.-P.F.); 2 Emergency Department, Hôpital Lariboisière, Assistance Publique-Hôpitaux de Paris, 75010 Paris, France; anthony.chauvin@aphp.fr; 3 Emergency Department, Hôpital Bichat, Assistance Publique-Hôpitaux de Paris, 75018 Paris, France; danielaiham.ghazali@aphp.fr; 4 Emergency Department, Hôpital Saint-Antoine, Assistance Publique-Hôpitaux de Paris, 75012 Paris, France; youri.yordanov@aphp.fr; 5 Emergency Department, Hôpital André Mignot, 78300 Versailles, France; aude.arsicaud@gmail.com; 6 Hôpital Henri Mondor, Assistance Publique-Hôpitaux de Paris, 94000 Créteil, France; pauline.gilleron@aphp.fr; 7 Hôpital Beaujon, Assistance Publique-Hôpitaux de Paris, 92024 Clichy, France; sonja.curac@aphp.fr; 8 Hôpital Pitié Salpetrière, Assistance Publique-Hôpitaux de Paris, 75013 Paris, France; Marie-caroline.richard@aphp.fr; 9 Hôpital Européen Georges Pompidou, Assistance Publique-Hôpitaux de Paris, 75015 Paris, France; anne-laure.feral-pierssens@aphp.fr; 10 Hôpital Cochin, Assistance Publique-Hôpitaux de Paris, 75014 Paris, France; barbara.villoing@aphp.fr; 11 Hôpital Ambroise Paré, Assistance Publique-Hôpitaux de Paris, 92100 Boulogne, France; sebastien.beaune@aphp.fr; 12 Hôpital Tenon, Assistance Publique-Hôpitaux de Paris, 75020 Paris, France; helene.goulet@aphp.fr; 13 Etablissement Français du Sang Ile de France, 75010 Paris, France; anne.francois@efs.sante.fr; 14 Etablissement Français du Sang Ile de France, Hôpital Henri Mondor, Assistance Publique-Hôpitaux de Paris, Université Paris Est Créteil, INSERM U955, Institut Mondor de Recherche Biomédicale, 94000 Créteil, France; france.pirenne@efs.sante.fr

**Keywords:** transfusion, red blood cell, blood product, anemia, hemoglobin, emergency department

## Abstract

Background: We aimed to describe red blood cell (RBC) transfusions in the emergency department (ED) with a particular focus on the hemoglobin (Hb) level thresholds that are used in this setting. Methods: This was a cross-sectional study of 12 EDs including all adult patients that received RBC transfusion in January and February 2018. Descriptive statistics were reported. Logistic regression was performed to assess variables that were independently associated with a pre-transfusion Hb level ≥ 8 g/dL. Results: During the study period, 529 patients received RBC transfusion. The median age was 74 (59–85) years. The patients had a history of cancer or hematological disease in 185 (35.2%) cases. Acute bleeding was observed in the ED for 242 (44.7%) patients, among which 145 (59.9%) were gastrointestinal. Anemia was chronic in 191 (40.2%) cases, mostly due to vitamin or iron deficiency or to malignancy with transfusion support. Pre-transfusion Hb level was 6.9 (6.0–7.8) g/dL. The transfusion motive was not notified in the medical chart in 206 (38.9%) cases. In the multivariable logistic regression, variables that were associated with a higher pre-transfusion Hb level (≥8 g/dL) were a history of coronary artery disease (OR: 2.09; 95% CI: 1.29–3.41), the presence of acute bleeding (OR: 2.44; 95% CI: 1.53–3.94), and older age (OR: 1.02/year; 95% CI: 1.01–1.04). Conclusion: RBC transfusion in the ED was an everyday concern and involved patients with heterogeneous medical situations and severity. Pre-transfusion Hb level was rather restrictive. Almost half of transfusions were provided because of acute bleeding which was associated with a higher Hb threshold.

## 1. Introduction

Blood products are scarce and valuable treatments that rely entirely on donors and should be administered judiciously. Moreover, improving transfusion practices may reduce the risk of adverse events such as pulmonary edema, hemolytic reactions, anaphylaxis, alloimmunization, and infection, among others. For this purpose, restrictive thresholds of hemoglobin (Hb) levels are favored over liberal thresholds [1].

In 2014, the French National Authority for Health published guidelines for red blood cell (RBC) transfusion [2]. These recommendations defined RBC transfusion thresholds in limited contexts and settings such as anesthesiology, intensive care, and onco-hematology. Basically, a threshold of 7 g/dL was recommended in the intensive care setting, in particular for patients with acute gastro-intestinal bleeding. This threshold could be increased to 10 g/dL in cases of acute coronary syndrome or heart failure or in a patient with poor tolerance of anemia (e.g., tachycardia, altered mental status, hypotension, dyspnea). In patients with solid or hematological malignancies likely to have chronic anemia due to chemotherapy, chronic inflammation, or bone-marrow infiltration, the threshold was 8 g/dL and 10 g/dL in cases of poor tolerance. In elderly patients above 80 years of age, the threshold was 7 g/dL, and 8 g/dL in case of history of heart failure or coronary artery disease, or 10 g/dL in cases of poor tolerance. These thresholds could be increased in case of rapid onset of anemia.

More recently, an international consensus conference established evidence-based recommendations for RBC transfusion thresholds for adults [3]. Authors also recommended a restrictive RBC transfusion threshold of 7 g/dL in critically ill but clinically stable intensive care patients, and of 7–8 g/dL for hemodynamically stable patients with acute gastrointestinal bleeding although the quality of evidence was moderate to low. The lack of evidence did not allow the expert panel to recommend any threshold for patients with lower intestinal or non-gastro-intestinal acute bleeding, onco-hematologic or coronary heart diseases.

Very few studies were focused on RBC transfusion in the emergency department (ED). The majority of them focused on critically ill patients in the intensive care unit (ICU) or on specific situations such as gastro-intestinal bleeding or trauma [4,5,6,7,8,9,10,11].

In this study, we aimed at describing patients that were transfused with RBC in the ED with a particular focus on the Hb level thresholds used in this setting.

## 2. Methods

### 2.1. Objectives

The objectives of this study were to assess the pre-transfusion Hb level depending on patients’ characteristics and determine which clinical characteristics were associated with a higher transfusion threshold in the ED.

### 2.2. Study Design, Settings, and Participants

We conducted an observational, multicenter, cross-sectional study in 12 EDs. All the centers were academic except one, and were located in, or near, Paris, France. The Etablissement Français du Sang (EFS) provided the list of all adult patients that received RBC transfusion, with the number of units, in those EDs from 1 January to 28 February 2018. The EFS is a public national organization that manages the collection, processing, and distribution of blood products, and guarantees the safety of the transfusion chain. Then we collected standardized data from patients’ medical files in each center, including demographic data (age, sex); medical history and usual treatments; reasons for ED referral (some patients reported several symptoms); vital parameters at triage including Hb point-of-care testing (POCT) HemoCue if performed; life-threatening conditions (shock, dyspnea or altered mental status according to the attending emergency physician); acute bleeding in the ED; pre-transfusion Hb level, reticulocyte count at arrival and post-transfusion Hb level based on blood withdrawal; number of RBC packs transfused in the ED; adverse events related to the transfusion; length of hospital stay and status at hospital discharge (deceased or alive).

Bleeding risk included personal medication, low platelet count (<50,000/mm^3^), and digestive causes.

Hematological diseases included hematological malignancies and non-malignant diseases such as idiopathic thrombocytopenia and sickle cell disease.

Overtransfusion was defined by a post-transfusion Hb level ≥ 10 g/dL.

Patients were categorized into four different groups: upper gastrointestinal bleeding, lower gastrointestinal bleeding, non-gastrointestinal bleeding, and absence of bleeding.

### 2.3. Analysis

Descriptive statistics were reported, namely median with interquartile range (IQR) for continuous variables and number with percentage (taking into account missing data) for categorical variables with comparison based on the Mann Whitney test or the chi square test, respectively. Due to the retrospective design, some data were missing from the ED medical files.

To assess the concordance between the measure of pre-transfusion Hb by the Hb POCT at arrival and the laboratory complete blood count (CBC), we plotted a Bland and Altman graphic and computed the percentage error. The reticulocyte counts were compared depending on the presence or absence of acute bleeding observed in the ED. Pre-transfusion Hb level was plotted depending on the presence or absence of acute bleeding or a life-threatening condition. In order to assess variables that were independently associated with a higher pre-transfusion Hb level, we first performed a bivariate analysis where the dependent variable was a pre-transfusion Hb ≥ 8 g/dL. Then, variables that were associated to the dependent variable with a *p*-value ≤ 0.1 were included in a multivariable logistic regression. In order to adjust the for patient’s severity, the variables that referred to life-threatening conditions (such as shock, dyspnea, altered mental status) and tachycardia on arrival were forced into the model whatever their *p*-value was. A backward selection was performed and non-significant variables were removed. All *p*-values were two-sided, with values of 0.05 or less considered as statistically significant. Data were analyzed with R 3.5.0 software (the R Foundation for Statistical Computing, Vienna, Austria).

### 2.4. Ethical Aproval

The study was approved by the Institutional Review Board of the Comité d’Evaluation de l’Ethique des projets de Recherche Biomédicale (CEERB) Paris-Nord n° 2019-023.

## 3. Results

### 3.1. General Characteristics, Reasons for ED Referral, and Physical Examination

During the study period, 529 patients received RBC transfusion in the 12 participating EDs. That is, on average, 0.7 patients per day and per center. Those patients received 1101 RBC packs. The number of RBC packs transfused in the EDs accounted for 4.4% of the total RBC transfused in the participating hospitals during the study period (25,567 RBC packs). General characteristics are presented in Table 1. The median age was 74 (59–85) years and 277 (52.4%) were females. The patients attended EDs during night shift (between 6 p.m. and 8 a.m.) in 185 (35.4%) cases and were referred to the ED by a non-emergency physician in 289 (55.3%) cases; mostly general physicians, laboratories, or nursing homes. When considering their medical history, 185 (35.2%) patients had active or an history of cancer or hematological disease. Anemia was chronic and secondary to vitamin or iron deficiency, chronic digestive or gynecological bleeding, renal insufficiency or malignancy with iterative transfusion support in 191 (40.2%) cases (54 missing data). Patients were considered at risk of bleeding in relation to medications in 238 (45.5%) cases, thrombopenia (<50,000/mm^3^) in 63 (12.9%) cases, and chronic gastro-intestinal lesion in 83 (15.8%) cases.

The main reasons for ED referral are summarized in Table 2. The two most frequent reasons were anemia in 191 (36.1%) cases and bleeding in 187 (35.3%) cases. Acute bleeding was actually observed in the ED for 242 (44.7%) patients, among which 145 (59.9%) were of gastrointestinal origin.

Patients had a life-threatening condition (dyspnea, shock or altered mental status) in 158 (30.2%) cases, were tachycardic on arrival in 169 (32.6%) cases or had a history of coronary artery disease in 144 (27.5%) cases.

**Figure 1:**
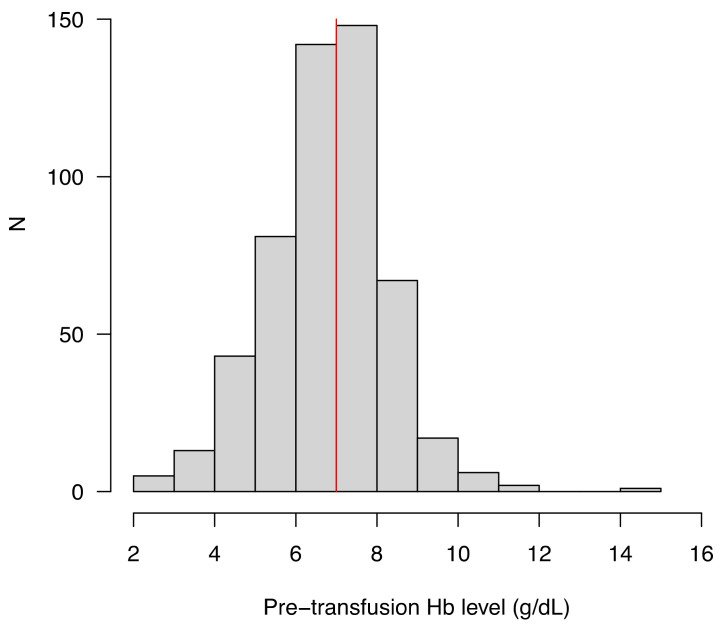
Histogram showing the distribution of the Hb level before transfusion (*N* = 529).

Pre-transfusion Hb level was 6.9 (6.0–7.8) g/dL (distribution is shown in Figure 1). The pre-transfusion Hb level (g/dL) was (7–8) for 119 (22.5%) patients, (8–9) for 78 (14.7%) patients, (9–10) for 17 (3.2%) patients, and ≥ 10 g/dL for 11 (2.1%) patients.

**Figure 2:**
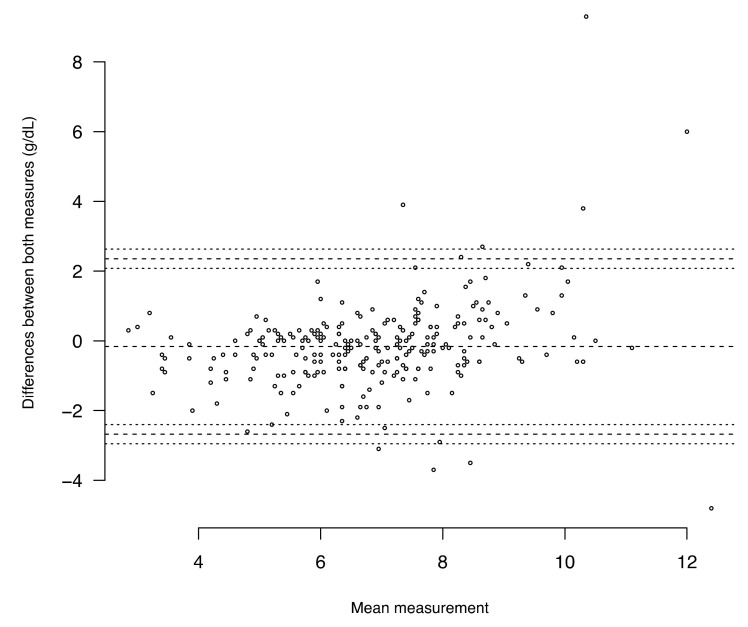
Concordance between pretransfusion Hb level (g/dL) depending on the measurement (point-of-care testing HemoCue vs. laboratory complete blood count with Bland and Altman graphic (*N* = 253). Each dot shows the difference between the 2 measurements for a patient over the mean of these 2 values. Dashed lines are the mean of the differences and the limits of agreement (2 standard deviations) above and below that. (The mean of the differences was −0.16 g/dL and the 2 SD were 2.35 g/dL and −2.68 g/dL.) Dotted lines are the 95% confidence intervals of each limit of agreement (2.63; 2.08; −2.40; −2.95).

**Figure 3:**
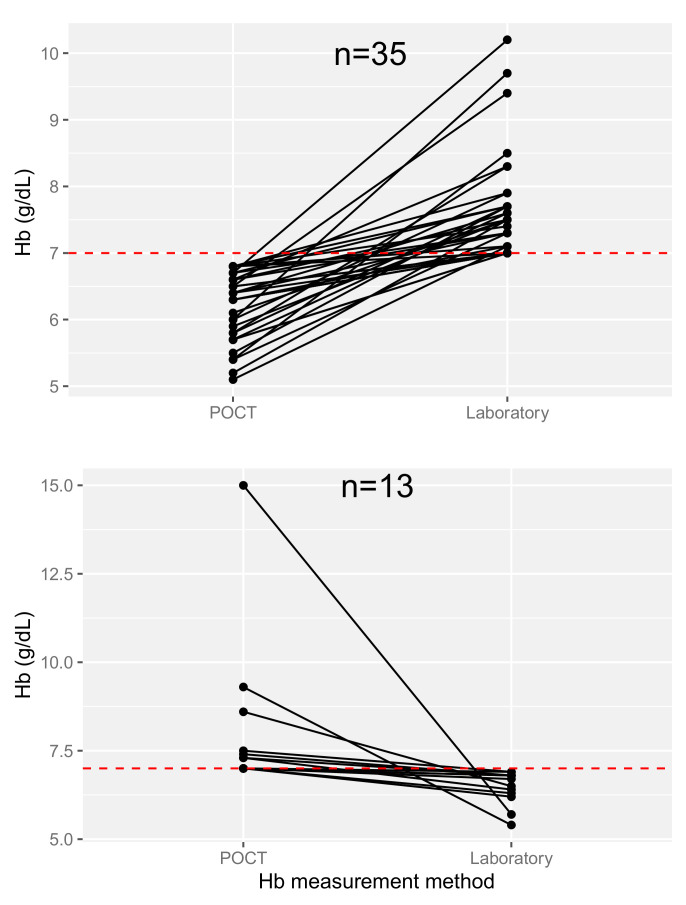
Discordances between pretransfusion Hb level (≥ or <7 g/dL) depending on the measurement method (point-of-care testing vs. laboratory complete blood count).

At arrival, a Hb POCT was performed for 253 (47.8%) patients among which 119 (47%) were presenting acute bleeding. While Figure 2 seems to show a good concordance of the pre-transfusion Hb level between both measures (Hb POCT vs. laboratory CBC), the percentage error was 37%. Twenty-two (8.7%) patients had a difference ≥ 2 g/dL between both measures. However, in 35 (13.8%) cases, the Hb level was below 7 g/dL with POCT while it was above with laboratory CBC, and it was the opposite for 13 (5.1%) patients (Figure 3).

**Figure 4:**
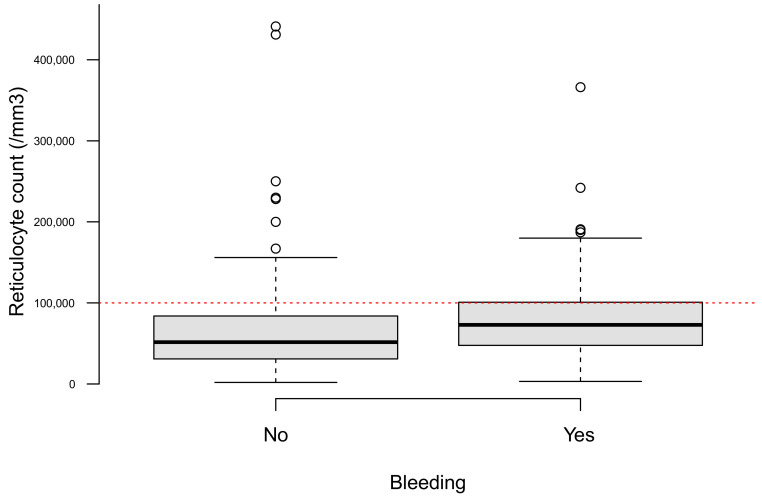
Boxplots showing the reticulocyte count depending on whether a bleeding was observed in the ED (*N* = 251).

A reticulocyte count was performed in 251 (47.4%) patients. Reticulocytes were 73,000 (47,282–101,000)/mm^3^ when bleeding was observed, whereas they were 51,700 (31,000–84,000)/mm^3^ in the absence of bleeding (Figure 4), *p* < 0.001.

### 3.2. Transfusion

**Figure 5:**
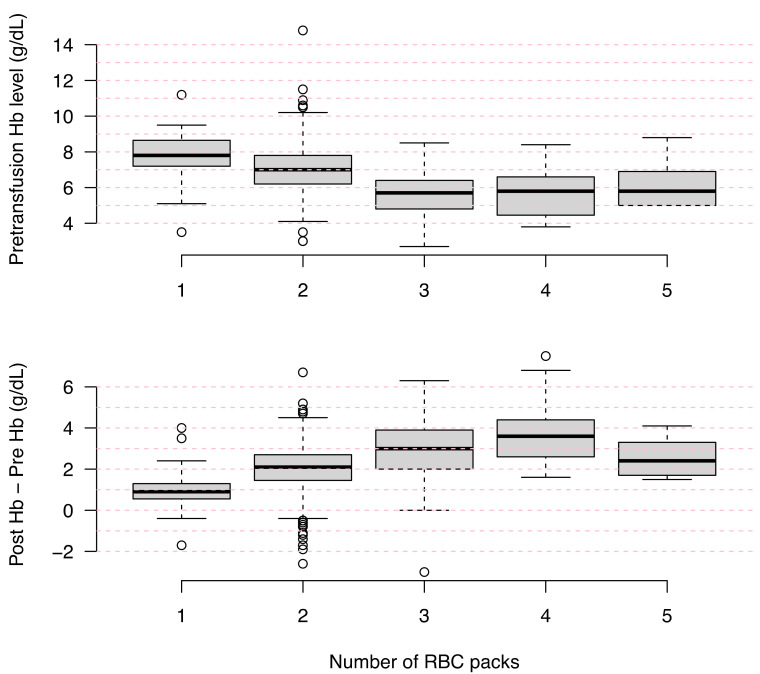
Boxplots of the pretransfusion Hb level and the Hb difference before and after transfusion depending on the number of transfused red blood cell packs in the ED (*N* = 512).

Patients received 1 RBC pack in 72 (14.1%) cases, 2 packs in 333 (65%) cases, 3 packs in 70 (13.7%) cases, and more than 3 packs in 37 (7.2%) cases (17 were missing data). After ED transfusion, Hb was obtained for 453 (85.6%) patients and was 8.9 (8.1–9.8) g/dL. Figure 5 shows the pretransfusion Hb level and the transfusion yield (difference of Hb level after and before transfusion) depending on the number of transfused RBC packs.

One hundred and three patients (19.5%) were overtransfused (post-transfusion Hb level ≥ 10 gr/dL). Those patients more frequently had coronary artery disease, hypertension, and chronic heart failure than the whole cohort and received 2 RBC packs in 68 (68%) and more than 2 RBC packs in 22 (22%) cases (Table 3).

**Figure 6:**
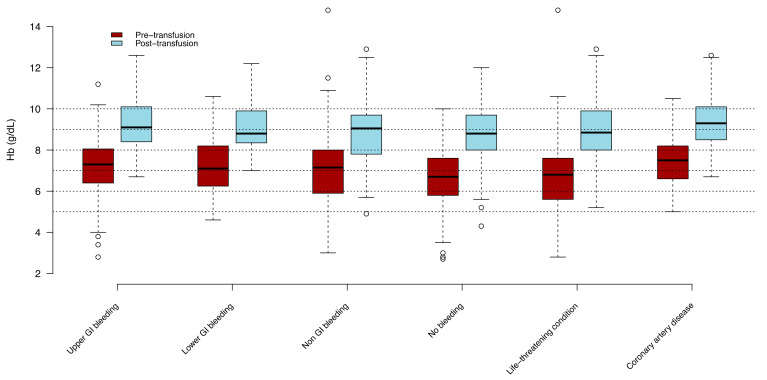
Boxplots of the pre-transfusion and post-transfusion Hb level in patients depending on the presence or absence of acute bleeding in the ED, a life-threatening condition (shock, dyspnea, altered mental status), and a history of coronary artery disease.

The pre-and post-transfusion Hb levels were similar regardless of the presence of a life-threatening condition, a history of coronary artery disease and whether acute bleeding was observed in the ED (Figure 6, Supplementary Table S1).

The emergency physician justified the need for urgent transfusion by the presence of signs of poor tolerance (dyspnea, shock, altered mental status, dizziness, fatigue) in 198 (37.4%) cases, the presence of active bleeding in 72 (13.6%) cases, a history of coronary artery disease in 19 (3.6%) cases, the rapid onset of anemia in 14 (2.6%) cases, post chemotherapy aplasia in 13 (2.5%) cases, and a low pre-transfusion Hb level in 7 (1.3%) cases. The transfusion motive was not notified in the ED medical chart for 206 (38.9%) patients. Among the 225 patients with a pre-transfusion Hb level ≥ 7 g/dL, only 33 (14.7%) were at low risk.

The results of the bivariate and the multivariate analysis are presented in Supplementary Table S2. In the multivariable logistic regression, after adjusting for patient clinical severity, variables that were associated with a higher pre-transfusion Hb level (≥8 g/dL) for transfusion were a history of coronary artery disease (OR: 2.09; 95% CI: 1.29–3.41), the presence of acute bleeding in the ED (OR: 2.44; 95% CI: 1.53–3.94), and an older age (OR: 1.02/year; 95% CI: 1.01–1.04).

### 3.3. Adverse Events

Eight (1.5%) adverse events were reported in the hospital medical files, among which were six cases of pulmonary edema, one case of hemolysis, and one episode of high blood pressure. Patients that experienced pulmonary edema after RBC transfusion were ≥63 years old, had a history of hypertension and had mostly a life-threatening condition (Supplementary Table S3). None of these patients were admitted to the ICU or died during hospital stay.

### 3.4. Outcomes

After visiting the ED, 448 (84.7%) were admitted to the ED short stay unit. Among those patients, 130 (29%) stayed less than 24 h before being discharged home. Other patients were admitted to wards in 49 (9.3%) cases, to the operating room in 1 (0.2%) case, and transferred to another hospital in 2 (0.4%) cases (1 was missing data). Forty-nine (9.3%) were admitted to the ICU during hospital stay, among which 28 (57.1%) were admitted directly from the ED. The length of hospital stay was 4 (1–11) days. At hospital discharge, 335 (77.2%) patients were discharged home, 76 (17.5%) were transferred to another hospital and 21 (4.8%) were deceased (97 missing data).

## 4. Discussion

In our study, a patient was transfused with RBC in the ED every one or two days. Acute bleeding was observed in half of the cases, 30% had a life-threatening condition, and 40% had chronic anemia. The transfusion motive was not notified in the ED medical chart for almost 40% of the patients. Pre-transfusion Hb level was 6.9 g/dL in median and was similar regardless of clinical stability or whether or not acute bleeding was observed. Acute bleeding, a history of coronary artery disease, and older age were associated with a higher pre-transfusion Hb level (≥8 g/dL) for transfusion.

If approximatively 1500 patients are transfused each day in France, the prevalence of RBC transfusion in the ED is not known. Langlais et al. assessed the appropriateness of RBC transfusion in the ED of an academic hospital in France before and after the implementation of a protocol [12]. Over one year, 228 patients were transfused, which was similar to the number of transfused patients in our study. It is possible that the prevalence of RBC transfusions in non-academic EDs differs from what we observed, and also may depend on some local specific organization, specifically for oncologic patients needing iterative transfusions. If the prevalence of RBC transfusions in the ED seems low, it is important to realize that it is a daily issue. Furthermore, this procedure can be extremely time-consuming for nurses and needs close monitoring to avoid adverse events. Other studies in the USA, UK, and Spain showed similar numbers of daily transfused patients in the ED [13,14,15,16,17].

Bleeding seems to be the main reason for transfused patients to seek emergency care as almost half of the patients had acute bleeding observed in the ED. In other studies focusing on ED transfusion, gastro-intestinal bleeding was one of the most frequent indication for RBC transfusion in the ED [12,13,14,15,17,18]. Contrarily to some other studies [13,19], bleeding due to trauma was scarce in our study. This may be explained by the French pre-hospital emergency care organization where a Mobile Intensive Care Unit, staffed by an emergency physician, can be sent on scene for pre-hospital medical assistance when a life-threatening condition is suspected. Thus, patients with major trauma are transported directly to the ICU without going through the ED.

Many of these transfusions occurred for patients with chronic diseases such as cancer, hematological disease, and iron or vitamin deficiency. Cancer exposes patients to multiple complications that can make them seek emergency care [20]. Some of these complications, such as bleeding, inflammation, cytotoxic chemotherapy, or bone marrow infiltration, may cause anemia needing transfusion. In other studies focusing on RBC transfusion in the ED, patients with cancer and hematological disease ranged from 20% to 45% [12,15,18,19,21,22]. In our study, patients were frequently admitted for less than 24 h raising the question of the appropriate use of ED for transfusion motive. Furthermore, Barr et al. showed that patients being treated for cancer had a higher risk of inappropriate transfusion in the ED with higher Hb thresholds [18].

It is very difficult to assess retrospectively the appropriateness of RBC transfusion but it seems that the pre-transfusion Hb levels used in the ED were rather low and in accordance with the restrictive thresholds recommended by the French guidelines [2]. However, assessing appropriateness only based on the threshold may not be relevant because in the emergency setting, those thresholds are often balanced by the clinical context such as hemodynamic instability, the acuteness and tolerance of anemia or, in case of acute bleeding, the importance of blood loss [23]. Furthermore, the patient Hb level is not known at arrival to the ED or may be falsely normal in the first hours of acute bleeding. Thus, an emergency physician may decide to transfuse a critically ill patient based only on physical examination. Surprisingly, in our study, the thresholds did not seem to be influenced by the clinical context and were similar whether or not there was acute bleeding or severity signs. In addition, deciding whether to transfuse a clinically stable patient is not a binary decision based solely on the Hb level but depends sometimes on subjective signs such as the tolerance of anemia. Patients may not have obvious life-threatening conditions or tachycardia but report extreme fatigue or exertional dyspnea that could justify transfusion, even above recommended thresholds, especially in elderly patients or in patients with coronary artery disease. Two studies assessed the appropriateness of RBC transfusion in the ED and showed a rate of inappropriate transfusions over 20% [17,18]. In these studies, patients with a higher pre-transfusion Hb level, chronic anemia, that were being being treated for cancer, or that were younger carried a higher risk of inappropriate transfusion, as well as treatment taking place in small non-teaching hospitals and the prescribing physicians having lower expertise. Thus, implementing transfusion protocols in the ED may reduce the rate of those inappropriate transfusions [16,19].

Another interesting finding was that almost 20% of the patients seemed to have been overtransfused with a post-transfusion Hb level ≥ 10 gr/dL. Given the number of RBC packs that those patients received, maybe a more graduated transfusion strategy (e.g., prescribing RBC packs one by one, instead of two or more from the beginning) could have been more appropriate and reduced the risk of overtransfusion, even if it is difficult to ascertain it retrospectively.

Among other findings, we showed that the Hb POCT results at ED arrival appeared to be concordant with the pre-transfusion Hb levels measured by laboratory CBC. However, when regarding an Hb level threshold of 7 d/dL, results were discordant for 48 (19%) patients. This is consistent with other studies in the ED [24,25,26]. Furthermore, the percentage error of 37% was above the admitted threshold of 30% which is defined a priori to indicate clinically useful agreement [27]. The reticulocyte count was significantly higher when acute bleeding was observed. Nevertheless, the difference between both groups was small with an important distribution overlap.

If adverse events seemed relatively scarce in the ED, the number of pulmonary edemas was higher than those reported in France by the EFS in 2018; 10 for 100,000 RBC packs transfused. On one hand, those patients that experienced pulmonary edema were mostly aged and with a history of hypertension but, on the other hand, they seemed to need urgent transfusion in the light of their low pre-transfusion Hb level and their risk category. However, transfusion must be cautiously given to elderly patients with a history of chronic cardiac diseases.

### Limitations

Our study has several limitations. First, we were unable to assess retrospectively the appropriateness of RBC transfusions because almost 40% of the patients had no justification to the transfusion in the ED medical file. Thus, deducing the need for urgent transfusion based only on the data from the ED medical file seemed hazardous. In addition, the retrospective data collection certainly introduced some interpretation bias. Secondly, our study enrolled all the patients that were transfused with RBC in the participating EDs but did not consider patients with anemia that were not transfused. Therefore, interpreting the pre-transfusion Hb level according to recommendations may be spurious because we cannot say how many patients that were not transfused should have been.

## 5. Conclusions

In our study, RBC transfusion in the ED was an everyday concern and involved patients with heterogeneous medical situations and severity. Therefore, the Hb thresholds recommended by the guidelines may be difficult to apply in the ED advocating for specific recommendation in this setting. Nevertheless, the pre-transfusion Hb level was rather restrictive and acute bleeding, a history of coronary artery disease, and older age seemed to be associated with a higher pre-transfusion Hb level (≥8 g/dL). A prospective larger study including patients attending ED for transfusion but also with anemia without being transfused is warranted.

## Figures and Tables

**Table 1:** General characteristics of patients transfused with red blood cells in the ED.

Variable			Missing Data
***N***	529		
**Female gender, *n* (%)**	277	(52.4)	0
**Age (years), median (IQR)**	74	(59–85)	0
**Night shift (6 p.m.–8 a.m.), *n* (%)**	185	(35.4)	6
**Medical history, *n* (%)**			
None	72	(13.7)	4
Hypertension	235	(44.8)	4
Coronary artery disease	144	(27.5)	4
Chronic heart failure	74	(14.1)	3
Chronic pulmonary disease	55	(10.4)	2
Renal insufficiency	18	(3.4)	4
Iron or vitamin deficiency	97	(18.3)	0
Solid malignancy	109	(20.7)	3
Hematological malignancy	69	(13.4)	4
Non-malignant hematological disease	15	(2.9)	4
**Chemotherapy < 1 month, *n* (%)**	62	(12.0)	12
**Iterative transfusions *n* (%)**	73	(14.1)	13
**Bleeding risk, *n* (%)**			
**Medication**	238	(45.6)	7
Antiplatelet therapy	135	(25.9)	
Vitamin K antagonist	48	(9.2)	
Direct oral anticoagulant	38	(7.3)	
Heparin	36	(6.9)	
Non-steroidal anti-inflammatory drug	8	(1.5)	
**Thrombopenia**	63	(12.9)	40
Other *	83	(15.8)	3
**Patient referred by, *n* (%)**	289	(56.0)	13
General practitioner	97	(18.8)	
Laboratory	72	(14.0)	
Nursing home care institution	50	(9.7)	
Pre-hospital emergency service	26	(5.0)	
Specialist	17	(3.3)	
Other	14	(2.7)	

* Angiodysplasia, peptic ulcer, esophageal varices, recent surgery.

**Table 2:** Reasons for ED referral and clinical exams at arrival of patients transfused with red blood cells in the ED.

Variables			Missing Data
***N***	529		
**Reasons for ED referral, *n* (%)**			0
Anemia	191	(36.1)	0
Bleeding	187	(35.3)	0
Fatigue	156	(29.5)	0
Dizziness	39	(7.4)	0
Thoracic pain	37	(7.0)	0
Trauma	32	(6.0)	0
Neurological disorders	18	(3.4)	0
**Physical examination, *n* (%)**			
Tachycardia (≥100/bpm)	169	(32.6)	10
Dyspnea	101	(19.3)	6
Shock	57	(10.9)	4
Altered mental status	32	(6.1)	5
**Any bleeding observed in the ED, *n* (%)**	242	(46.0)	3
Gastro-intestinal	145	(27.6)	3
Cutaneous	29	(5.5)	3
Mucosal *	22	(4.2)	3
Genitourinary	21	(4.0)	3
Urinary	17	(3.2)	3
Peritoneal	3	(0.6)	3
Hemoptysis *	3	(0.6)	3

* One patient had both mucosal and hemoptysis.

**Table 3:** Characteristics of patients that were overtransfused with red blood cells (post-transfusion Hb level ≥ 10 gr/dL) in the ED.

Variable			Missing Data
***N***	103		
**Female gender, *n* (%)**	63	(61.2)	0
**Age (years), median (IQR)**	80	(66–88)	0
**Coronary artery disease, *n* (%)**	40	(39.2)	1
**Hypertension, *n* (%)**	52	(51.0)	1
**Chronic heart failure, *n* (%)**	22	(21.6)	1
**Chronic anemia, *n* (%)**	34	(40.5)	19
**Malignancy, *n* (%)**	36	(35.6)	2
**Hb POCT, median (IQR)**	7.5	(6.5–9.0)	46
**Tachycardia (≥100/bpm), *n* (%)**	32	(32.0)	3
**Life-threatening condition, *n* (%)**	8	(31.1)	0
**Any bleeding observed in the ED, *n* (%)**	8	(50.5)	0
**Pre-transfusion Hb level, median (IQR)**	7.8	(6.9–8.6)	1
**Number of RBC pack, *n* (%)**			3
1	10	(10.0)	
2	68	(68.0)	
3	9	(9.0)	
4	12	(12.0)	
5	1	(1.0)	
**Adverse event, *n* (%)**	1	(1.0)	0
**ICU during hospital stay, *n* (%)**	9	(10.2)	15
**Length of hospital stay, median (IQR)**	2	(1–8)	29
**Hospital death, *n* (%)**	1	(1.1)	15

Hb hemoglobin, GI gastro-intestinal, ICU intensive care unit, POCT point-of-care testing, RBC red blood cell.

## Data Availability

The data presented in this study are available on request from the corresponding author.

## Supplementary Materials

The following are available online at https://www.mdpi.com/article/10.3390/jcm10112475/s1, Table S1: Pre- and post-transfusion hemoglobin (Hb) level in the ED (median [interquartile range] g/dL) depending on the bleeding group, the presence of a life-threatening condition (shock, dyspnea, or altered mental status) or a history of coronary artery disease, Table S2: Bivariate and multivariate analyses to identify variables that were associated with a higher threshold for transfusion (pre-transfusion Hb level ≥ 8 g/dL). In order to adjust for patient severity, the variables that referred to life-threatening conditions and tachycardia on arrival were forced into the model, Table S3: Characteristics of the 6 patients that experienced pulmonary edema after ED RBC transfusion.
