# Supplementary material for: Red Blood Cell Transfusion in the Emergency Department: An Observational Cross-Sectional Multicenter Study
Source: J Clin Med. 2021 Jun 2;10(11):2475. doi: 10.3390/jcm10112475 (PMC8199757; doi:10.3390/jcm10112475)
Supplement: Supplementary file 1 [file jcm-10-02475-s001.zip › Supplementary Table 1.pdf]

Supplementary Table 1. Pre- and post-transfusion hemoglobin (Hb) level in the ED (median [interquartile range] g/dL) depending on the bleeding group, the presence of a life-threatening condition (shock, dyspnea, or altered mental status) or a history of coronary artery disease

**Hb level**	Upper GI bleeding (n=97)	Lower GI bleeding (n=48)	Non-GI bleeding (n=95)	No bleeding (n=284)	Life-threatening condition (n=158)	Coronary artery disease (n=144)
**Pre-transfusion**	7.3 [6.4-8.0]	7.1 [6.2-8.2]	7.1 [5.9-8.0]	6.7 [5.8-7.6]	6.8 [5.6-7.6]	7.5 [6.6-8.2]
**Post-transfusion**	9.1 [8.4-10.1]	8.8 [8.3-9.9]	9.0 [7.8-9.7]	8.8 [8.0-9.7]	8.8 [8.0-9.9]	9.3 [8.5-10.1]
