# Supplementary material for: Red Blood Cell Transfusion in the Emergency Department: An Observational Cross-Sectional Multicenter Study
Source: J Clin Med. 2021 Jun 2;10(11):2475. doi: 10.3390/jcm10112475 (PMC8199757; doi:10.3390/jcm10112475)
Supplement: Supplementary file 1 [file jcm-10-02475-s001.zip › Supplementary Table 2.pdf]

Supplementary Table 2. Bivariate and multivariate analyses to identify variables that were associated with a higher threshold for transfusion (pre-transfusion Hb level ≥ 8 g/dL). In order to adjust for patient severity, the variables that referred to life-threatening conditions and tachycardia on arrival were forced into the model.

	Bivariate			Multivariate		
Variable	OR	95% CI	p-value	OR	95% CI	p-value
**Male gender**	1.25	[0.82 to 1.92]	0.3			
**Age (/year)**	1.02	[1.01 to 1.04]	<0.001	1.02	[1.01 to 1.04]	0.007
**Night shift (6PM-8AM)**	0.9	[0.56 to 1.40]	0.6			
**Referred by a physician**	0.75	[0.48 to 1.15]	0.2			
**Reason for ED referral**						
Dyspnea	0.45	[0.24 to 0.80]	0.009			
Chest pain	1.3	[0.56 to 2.74]	0.5			
Bleeding	2.81	[1.82 to 4.36]	<0.0001			
Fatigue	0.6	[0.36 to 0.98]	0.05			
Dizziness	1.2	[0.52 to 2.52]	0.6			
Cytopenia	0.52	[0.31 to 0.83]	0.007			
Neurological disorders	0.78	[0.18 to 2.43]	0.7			
Trauma	2.55	[1.17 to 5.33]	0.01			
**History of**						
Chronic pulmonary disease	0.47	[0.18 to 1.04]	0.09			
Coronary artery disease	2.43	[1.55 to 3.81]	0.0001	2.09	[1.29 to 3.41]	0.003
Hypertension	1.86	[1.21 to 2.88]	0.005			
Chronic heart failure	2.79	[1.62 to 4.74]	0.0002			
Cancer or hemopathy	1.08	[0.69 to 1.68]	0.7			
**Bleeding risk medication***	1.71	[1.11 to 2.64]	0.01			
**Life-threatening condition**						
Dyspnea	0.53	[0.27 to 0.96]	0.05	0.57	[0.28 to 1.09]	0.1
Shock	1.23	[0.61 to 2.32]	0.5	1.00	[0.47 to 2.03]	0.9
Altered mental status	0.75	[0.25 to 1.84]	0.6	0.65	[0.20 to 1.73]	0.4
**Tachycardia (≥100 bpm)**	0.85	[0.53 to 1.34]	0.5	1.07	[0.64 to 1.77]	0.8
**Acute bleeding**	2.32	[1.50 to 3.62]	0.0001	2.44	[1.53 to 3.95]	<0.001

* Antiplatelet therapy, Vitamin K antagonist, Direct oral anticoagulant, Heparin, Non-steroidal anti-inflammatory drug
