# Supplementary material for: Red Blood Cell Transfusion in the Emergency Department: An Observational Cross-Sectional Multicenter Study
Source: J Clin Med. 2021 Jun 2;10(11):2475. doi: 10.3390/jcm10112475 (PMC8199757; doi:10.3390/jcm10112475)
Supplement: Supplementary file 1 [file jcm-10-02475-s001.zip › Supplementary Table 3.pdf]

Supplementary Table 3. Characteristics of the 6 patients that experienced pulmonary edema after ED RBC transfusion.

Patient	Age (years)	Medical history	Arterial BP at ED arrival	Acute bleeding	Transfusion justification	Life-threatening condition*	Pre-transfusion Hb level (g/dL)	Number of RBC packs
1	88	Hypertension	120/69	Non-GI bleeding	Poor tolerance	Yes	7.2	1
2	77	Missing data	169/78	No	Poor tolerance	Yes	8.9	1
3	96	Hypertension / Coronary vessel disease	136/52	No	Poor tolerance	Yes	6.7	2
4	81	Hypertension / Coronary vessel disease	189/68	No	Dyspnea	Yes	7.8	1
5	63	Hypertension	119/41	No	Missing	No	3.9	4
6	97	Hypertension / Coronary vessel disease	103/47	Non-GI bleeding	Hemorrhagic shock	Yes	5.1	3

BP blood pressure, ED emergency department, GI gastro-intestinal, Hb hemoglobin, RBC red blood cell

* Shock, dyspnea or altered mental status
